# Evening chronotype is associated with elevated biomarkers of cardiometabolic risk in the EpiHealth cohort: a cross-sectional study

**DOI:** 10.1093/sleep/zsab226

**Published:** 2021-09-04

**Authors:** Gabriel Baldanzi, Ulf Hammar, Tove Fall, Eva Lindberg, Lars Lind, Sölve Elmståhl, Jenny Theorell-Haglöw

**Affiliations:** 1 Department of Medical Sciences, Molecular Epidemiology and Science for Life Laboratory, Uppsala University, Uppsala, Sweden; 2 Department of Medical Sciences, Respiratory, Allergy and Sleep Research, Uppsala University, Uppsala, Sweden; 3 Department of Medical Sciences, Cardiovascular Epidemiology, Uppsala University, Uppsala, Sweden; 4 Department of Clinical Sciences, Malmö, Division of Geriatric Medicine, Lund University, Lund, Sweden; 5 CRC, Skåne University Hospital, Malmö, Sweden

**Keywords:** chronotype, sleep habits, proteomics, cohort studies, cardiovascular diseases, metabolic diseases

## Abstract

**Study Objectives:**

Individuals with evening chronotype have a higher risk of cardiovascular and metabolic disorders, although the underlying mechanisms are not well understood. In a population-based cohort, we aimed to investigate the association between chronotype and 242 circulating proteins from three panels of established or candidate biomarkers of cardiometabolic processes.

**Methods:**

In 2,471 participants (49.7% men, mean age 61.2 ± 8.4 SD years) from the EpiHealth cohort, circulating proteins were analyzed with a multiplex proximity extension technique. Participants self-reported their chronotype on a five-level scale from extreme morning to extreme evening chronotype. With the intermediate chronotype set as the reference, each protein was added as the dependent variable in a series of linear regression models adjusted for confounders. Next, the chronotype coefficients were jointly tested and the resulting *p*-values adjusted for multiple testing using a false discovery rate (5%). For the associations identified, we then analyzed the marginal effect of each chronotype category.

**Results:**

We identified 17 proteins associated with chronotype. Evening chronotype was positively associated with proteins previously linked to insulin resistance and cardiovascular risk, namely retinoic acid receptor protein 2, fatty acid-binding protein adipocyte, tissue-type plasminogen activator, and plasminogen activator inhibitor 1 (PAI-1). Additionally, PAI-1 was inversely associated with the extreme morning chronotype.

**Conclusions:**

In this population-based study, proteins previously related to cardiometabolic risk were elevated in the evening chronotypes. These results may guide future research in the relation between chronotype and cardiometabolic disorders.

**Statement of Significance**

A person’s chronotype is defined by diurnal preference as well as sleeping habits. Previous studies showed that individuals with later sleeping hours (i.e. later or evening chronotypes) have a higher risk for type 2 diabetes and cardiovascular disease. However, the mechanisms for this increased risk are not well understood. In this large population-based study, we investigated the association between chronotype and circulatory proteins relevant for cardiometabolic processes. We found that individuals with evening chronotypes had an increased level of proteins previously related to coronary heart disease and insulin resistance. Additionally, proteins levels increased progressively with greater evening preference. Our findings may guide future experimental and longitudinal studies to understand how evening chronotype connects to health problems.

## Introduction

The human sleep-wake cycle synchronizes with the day-night cycle through a number of external signals such as ambient light, food, and physical activity [1, 2]. Due to individual differences in the reaction to these external signals, different chronotypes exist. Individuals that sleep and wake up early are defined as early or morning chronotypes, while those who have a later bedtime and wakeup time are defined as late or evening chronotypes [3]. Notably, the biological differences between the chronotypes stretch beyond the sleep timing, involving differences in body temperature circadian phases, hormone secretion patterns, timing of alertness [4], and disease risk [5].

Observational studies show associations between evening chronotypes and adverse health outcomes, including cardiovascular disease (CVD) [6] and metabolic disorders [7]. In the National FINRISK Study (*n* = 6,258), evening-types had a higher odds (OR = 2.5, 95% CI = 1.5 to 4.4) of having type 2 diabetes (T2D) regardless of sleep duration and sufficiency [6]. Using UK Biobank data (*n* = 433,268), Knutson *et al.* found that evening-types have increased odds of several comorbidities, such as T2D, CVD, renal disease, and endocrine disorders [5].

Several explanations for the association between evening chronotype and cardiometabolic disorder have been suggested. One hypothesis is an unhealthier lifestyle, as later chronotypes show lower sports participation, later eating time, as well as more smoking and higher alcohol consumption [8]. Another explanation is that a difference in sleep timing between working and free days may lead to circadian misalignment, and thereby chronic sleep insufficiency, which has been related to health problems [9, 10]. Circadian misalignment can contribute to the development of obesity [10], while the adipose tissue can secrete a large array of active compounds with potential repercussions to cardiometabolic health. In addition, shift work can have deleterious impacts on health, especially when not aligned with the worker’s chronotype [11].

The molecular mechanisms that connect the evening chronotype to metabolic disorders are unknown and only a few studies have investigated the biomarkers profile in chronotypes. Two studies have investigated chronotype in relation to the metabolome and found that later sleep timing was associated with branched-chain amino acids (BCAA) and their gamma-glutamyl metabolites, as well as negatively correlated with acylcarnitines [12, 13]. Higher circulating levels of BCAA, as well as reduction in medium-chain acylcarnitines during oral glucose tolerance test, have been linked to insulin resistance [14, 15]. In addition, urinary carnitines were altered in early chronotype nurses working night shifts [16].

There is a lack of studies exploring the relationship of chronotype with circulating proteins biomarkers. The discovery of biomarkers associated with chronotypes has the potential to guide this research field toward important mechanisms behind the adverse health outcomes of later sleep timing. Therefore, the present study aims to identify plasma protein biomarkers associated with chronotypes in a large population-based cohort in Sweden.

## Methods

### Cohort description

**Figure 1.**
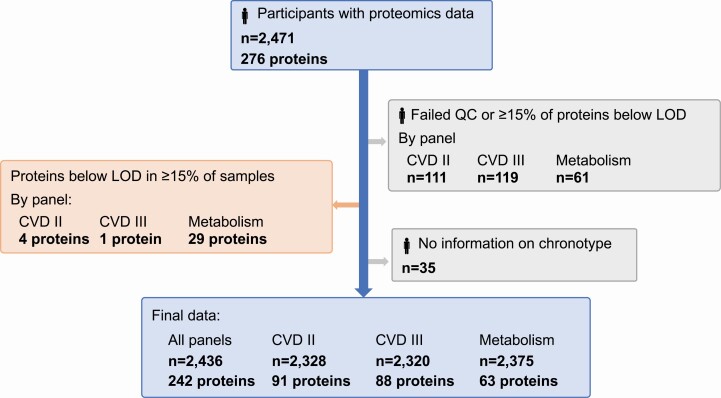
Flowchart of the study population in total and by Olink panels. Individuals were excluded from a panel if their sample failed Olink’s quality control or more than 15% of the proteins in that panel were below the lower limit of detection (LOD). Individuals were also excluded if they were missing information on chronotype. Proteins were excluded if they were below the LOD in more than 15% of the samples. Final study population comprised 2,436 individuals and 242 proteins. CVD, cardiovascular disease.

The Epidemiology for Health cohort (EpiHealth; https://www.epihealth.lu.se/) study started in 2011 through a consortium between Uppsala and Lund universities to study lifestyle factors, genotypes, and the development of degenerative disorders [17]. For recruitment, an equal number of male and female participants were invited across age and sex strata [17]. Participants (age 45–75, *n* = 25,080) answered an extensive online questionnaire and visited one of the test centers for blood sampling and anthropometric measuring. The current study included 2,471 participants who were amongst the first participants included in the Uppsala site and were also selected for genotyping [18] and plasma proteomic profiling. Individuals with proteomics data were not markedly different from the whole EpiHealth cohort [19], besides a somewhat higher proportion of males (49.7% vs. 43.7%). Individuals with no information on chronotype from the EpiHealth questionnaire (*n* = 35) were excluded and the final sample comprised 2,436 participants (Figure 1).

### Chronotype

Chronotype was assessed using the question: “Are you a morning person or an evening person?.” Answers were given on a scale from 1 to 5 (1 = extreme morning-type; 2 = moderate morning-type; 3 = intermediate; 4 = moderate evening-type; and 5 = extreme evening-type).

### Proteomic analysis

Blood samples were collected in the test center from December 2011 to December 2012. Sampling was performed during the day (between 7:00 am and 5:00 pm), after at least 6 h of fasting. After separation of plasma, samples were stored at −80°C (mean storage time = 4.5 ± 0.3 SD years; range 4.0–5.0 years).

Protein biomarkers were measured using the multiplex proximity extension assay (PEA) by Olink (Olink Proteomics, Uppsala, Sweden). Each PEA panel simultaneously quantifies 92 proteins using a pair of protein-specific antibodies attached to complementary oligonucleotides [20]. Proteins for the different panels are selected based on known or hypothesized links to disease groups. For the current study, the Olink panels Cardiovascular II, Cardiovascular III, and Metabolism (https://www.olink.com/resources-support/document-download-center/) were used.

Individuals were excluded from a panel if their sample failed Olink’s quality control or more than 15% of the proteins (i.e. more than 10 proteins) in that panel were below the lower limit of detection (LOD). Individuals excluded were not different in terms of age, sex, body mass index (BMI), waist circumference, and weight [21]. Additionally, proteins were excluded from the analysis if they were below the LOD in more than 15% of the samples. The final study sample comprised proteomic profile data for 2,328 individuals on 88 proteins from panel CVD II, for 2,320 individuals on 91 proteins from panel CVD III, and for 2,375 individuals on 63 proteins from panel Metabolism (Figure 1). Although samples were considered randomized on plates and data had been normalized, protein values were adjusted for storage time and plate through linear regression and the standardized residuals (*z*-score) were used as the outcome variables in the subsequent analyses.

### Covariates

**Figure 2.**
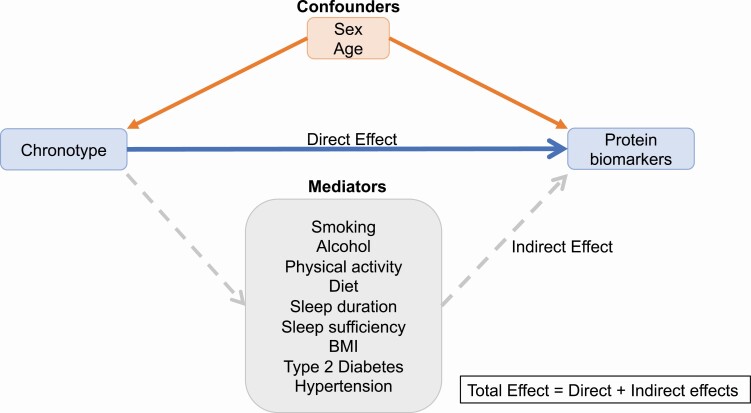
Simplified directed acyclic graph depicting the direct and indirect effects of chronotype on circulating protein biomarkers.

Relevant covariates were categorized according to a hypothetical causal diagram depicted in a directed acyclic graph (DAG) using the application DAGitty (www.dagitty.net; Supplementary Figure S1) [22]. Following the d-separation criteria, we identified the covariates categorized as confounders or mediators. Confounders were ancestors variables of both chronotype and proteins in a pathway that did not include chronotype. Potential mediators were defined as the covariates which were a descendent of the exposure and an ancestor of the outcome in the causal pathway from chronotype to circulating proteins levels (Figure 2) [22].

The confounders identified were sex and age at visit. Participants’ sex was indicated as either male or female.

The potential mediators identified were smoking, alcohol, physical activity, diet, sleep sufficiency, sleep duration, BMI, hypertension, and diabetes. Smoking was entered as a nominal variable (never, former, or current smoker) based on the questions “Do you smoke?” and “Have you ever smoked?.” Drinking habits were estimated using the modified version of the Alcohol Use Disorders Identification Test-Concise (AUDIT-C) [23]. Self-reported leisure-time physical activity was classified into four possible levels: a low level consisting of mainly sedentary activity; a medium-low level consisting of light intensity activity, such as walking and dancing; a medium-high level including regular exercises and ordinary house chores; and a high level including regular vigorous-intensity activities. Diet was assessed with the Healthy Nordic Food Index (HNFI), which was calculated from the food frequency questionnaire in the original cohort as described at Warensjö Lemming *et al.* [24]. The HNFI score ranges from 0 to 6 based on the consumption of six food groups: apples and pears, root vegetables, cruciferous vegetables, whole-grain bread, oatmeal, and fish. Insufficient sleep was assessed by the sleep sufficiency index (SSI) [25]. SSI was calculated by the ratio of reported sleep duration to reported sleep need. Individuals with an SSI < 0.8 were considered as having insufficient sleep [25]. Hypertension and diabetes were defined based on self-reported doctor diagnosis or medication use for the condition. BMI was calculated as weight/height [2] (kg/m^2^) based on the study site measurements. Fat mass was assessed using a bioimpedance scale (Tanita, Tokyo, Japan) [26]. Body fat percentage was calculated by the ratio of fat mass to weight.

Lastly, time of the day for blood sampling was added as a covariate of technical variation as samples were collected over a large time span and protein levels varied depending on the time of sampling.

### Statistical analysis

Chronotype was modeled as the independent variable with the intermediate chronotype set as the reference category, and protein biomarkers as the dependent variable. The effect of chronotype on each of the 242 proteins was investigated in two steps using a series of linear regressions. The first step was the main analysis and examined the total effect of chronotype by adjusting for the confounders’ age and sex and in addition sampling time. Significance was tested by applying a joint Wald test on the four chronotype coefficients. Adjustment for multiple comparisons was performed using the Benjamini–Hochberg method [27] on the joint test *p*-values with a false discovery rate (FDR) set at 5%. The second step examined the direct effect of chronotype on proteins. In this step, the potential mediators from the DAG were also included in the model with the aim of estimating the effect of chronotype that is not mediated by another covariate. The proteins that reached in the main analysis the threshold of an FDR *p*-value < 0.05 were sequentially added as the dependent variable in the model further adjusted for the potential mediators. In this step, a joint test *p*-value < 0.05 was considered significant. Finally, for the proteins identified in the first and second steps, we looked at the regression β-coefficients, 95% CIs, and *p*-values to determine how each chronotype category associated with the proteins. Also, for the proteins identified, we analyzed the regression residuals for approximate normality and homogeneity of variance.

To analyze the potential effect of sleep insufficiency on the relationship between chronotype and protein biomarkers, the proteins identified in step 1 were assessed in a total effect model with the addition of an interaction term between chronotype and insufficient sleep. Individuals with no information on SSI were excluded from this analysis (*n* = 109).

Four sensitivity analyses were performed. First, we repeated the analysis on the total effect of chronotype on circulating proteins after excluding shift workers (*n* = 124). Second, because some of the biomarkers identified had been previously related to obesity and fat tissue mass, we compared the results of the direct effect model after additional adjustment for waist-to-hip ratio and body fat percentage. Third, to better control the effect that certain conditions or medications could have on protein levels, the total effect of chronotype was further studied for the proteins identified in the main analysis by restricting the analysis to those participants without self-reported hypertension, diabetes, dyslipidemia, or medication use for these conditions. Finally, in the fourth sensitivity analysis, we compared the results of complete case analysis to the results produced from the imputed data.

All analyses were performed with the software Stata 15 (Stata Corp., Texas, USA). Figures with results were produced in the software R version 4.0.3 (2020-10-10).

### Imputation of missing variables

Variables with the most missing information were smoking (*n* = 463), alcohol (n = 148), SSI (*n* = 109), and physical activity (*n* = 26). Missing values were imputed with multiple imputations by 10 chained equations. To account for the uncertainty of imputations, analysis was performed on each of the completed datasets and then combined using Rubin’s combination rules [28]. Continuous variables were imputed using linear regression, binary variables using logistic regression, and categorical variables with > 2 levels using ordinal logistic regression. Variables used for imputation included all confounders, mediators, and protein variables, as well as body fat mass, waist-to-hip ratio, total energy intake, marital status, shift work, and snus use.

### Ethical considerations

Data collection for EpiHealth was approved by the Ethics Board in Uppsala, Sweden (Dnr 2010/402—December 1, 2010 and November 17, 2011). The Swedish Data Protection Authority also inspected the study and found it to be clear of objections (Dnr 307-2011—March 3, 2011). The current study was approved by the Ethics Boards in Uppsala (Dnr 2017/487—December 13, 2017).

## Results

The study population is described in Table 1. About half of the individuals were males (49.7%) and the mean age was 61.2 (±8.4 SD) years. Most of the participants described themselves as the intermediate chronotype with 845 (34.7%) participants, while 383 (15.7%) defined themselves as an extreme evening-type and 511 (21.0%) as an extreme morning-type. Extreme evening-types and extreme morning-types were slightly younger, more often single or living alone, and more often a current smoker than intermediate types. No significant differences were seen for diet (HNFI score), alcohol score, BMI, or sleep duration across the categories. There was, however, a larger proportion of participants with an SSI < 0.8 among the extreme evening-types.

**Table 1.** Study population characteristics (2,436 Swedish adults from the Uppsala site of EpiHealth cohort, age 45–75 years)

	Extreme morning n = 511	Moderate morning n = 391	Intermediate n = 845	Moderate evening n = 306	Extreme evening n = 383	
	Mean (SD) or n (%)	Mean (SD) or n (%)	Mean (SD) or n (%)	Mean (SD) or n (%)	Mean (SD) or n (%)	P
Male*	249 (48.7)	199 (50.9)	410 (48.5)	166 (54.2)	183 (47.8)	0.41
Age (years)	60.8 (8.3)	60.2 (8.2)	62.3 (8.4)	60.1 (8.7)	61.6 (8.1)	<0.001
Marital status						<0.001
Single/living alone	127 (24.9)	84 (21.5)	178 (21.1)	69 (22.5)	124 (32.6)	
Married/living together	384 (75.1)	306 (78.5)	667 (78.9)	237 (77.5)	256 (67.4)	
Education						0.066
Compulsory	107 (20.9)	71 (18.2)	162 (19.2)	49 (16.0)	67 (17.5)	
Secondary	143 (28.0)	99 (25.4)	209 (24.8)	67 (21.9)	87 (22.8)	
University	192 (37.6)	174 (44.6)	352 (41.8)	156 (51.0)	183 (47.9)	
Other	69 (13.5)	46 (11.8)	120 (14.2)	34 (11.1)	45 (11.8)	
Current smoker	44 (10.2)	20 (6.5)	52 (7.6)	25 (10.5)	40 (12.7)	0.034
AUDIT-C score	3.4 (1.8)	3.5 (1.6)	3.5 (1.7)	3.7 (1.8)	3.6 (1.9)	0.1
BMI (kg/m^2^)	26.3 (3.6)	26.2 (3.7)	26.4 (3.9)	26.7 (3.9)	26.9 (4.0)	0.055
WHR	0.89 (0.08)	0.90 (0.08)	0.89 (0.08)	0.90 (0.08)	0.91 (0.08)1)	0.17
Body fat (%)	30.1 (7.9)	29.7 (7.9)	30.5 (8.1)	29.9 (8.5)	31.2 (8.4)	0.099
Physical activity level						0.24
Low	30 (5.9)	15 (3.9)	37 (4.4)	17 (5.6)	29 (7.7)	
Medium-low	169 (33.4)	128 (33.3)	296 (35.4)	121 (39.5)	138 (36.5)	
Medium-high	212 (41.9)	165 (43.0)	364 (43.5)	117 (38.2)	155 (41.0)	
High	95 (18.8)	76 (19.8)	139 (16.6)	51 (16.7)	56 (14.8)	
HNFI score	2.7 (1.5)	2.8 (1.4)	2.8 (1.5)	2.6 (1.4)	2.8 (1.4)	0.36
Sleep duration (hours)	6.8 (1.0)	6.9 (0.9)	6.9 (1.0)	6.9 (0.9)	6.9 (1.0)	0.32
SSI	0.95 (0.13)	0.95 (0.12)	0.94 (0.13)	0.93 (0.12)	0.93 (0.14)	0.075
Insufficient sleep	65 (12.9)	45 (11.9)	119 (14.6)	53 (18.0)	77 (21.0)	0.002
Hypertension	142 (27.8)	103 (26.3)	219 (25.9)	71 (23.2)	115 (30.0)	0.32
Diabetes	15 (2.9)	14 (3.6)	39 (4.6)	4 (1.3)	16 (4.2)	0.087

Variables presented as mean (±SD) or as *n* (%).

To obtain *p*-values, ANOVA and chi-square test were used for continuous and categorical variables, respectively. AUDIT-C score, Alcohol Use Disorders Identification Test-Concise score; BMI, body mass index; HNFI score, Healthy Food Nordic index score; SSI, sleep sufficiency index; WHR, waist-to-hip ratio.

*Information on gender was not available for 22 participants.

### Association between chronotypes and protein biomarkers

**Figure 3.**
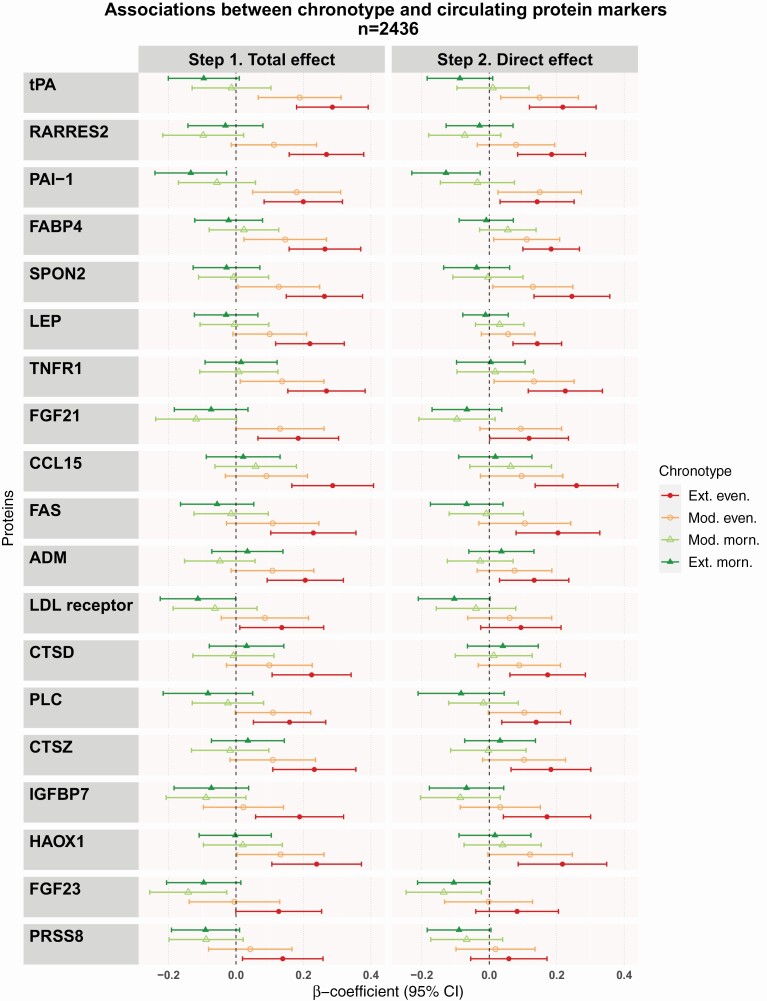
Associations between chronotype and circulating protein markers. Figure shows the results from the linear regression modelling of total or direct effect of chronotype. The total effects model was adjusted for age, sex, and sampling time. The direct effects model was further adjusted for smoking, alcohol, physical activity, diet, sleep sufficiency, sleep duration, BMI, hypertension, and diabetes. Results are presented as β-coefficients and 95% confidence intervals representing changes in protein measurement in relation to the intermediate chronotype. ADM, Adrenomedullin; CCL15, C-C motif chemokine 15; CTSD, cathepsin D; CTSZ, cathepsin Z; FAS, tumor necrosis factor receptor superfamily member 6; FABP4, fatty acid-binding protein, adipocyte; FGF21, fibroblast growth factor 21; FGF23, fibroblast growth factor 23; HAOX1, hydroxyacid oxidase 1; IGFBP7, insulin-like growth factor-binding protein 7; LEP, leptin; LDL receptor, low-density lipoprotein receptor; PAI-1, plasminogen activator inhibitor 1; PLC, perlecan; PRSS8, prostasin; RARRES2, retinoic acid receptor protein 2; SPON2, spondin-2; tPA, tissue-type plasminogen activator; TNFR1, tumor necrosis factor receptor 1; Ext, extreme; Even, evening-type; Mod, moderate; Morn, morning-type.

We identified 19 proteins associated with chronotype in the first step (model adjusted for age, sex, and sampling time) after adjusting for multiple testing (Figure 3 and Supplementary Table S1). All but two proteins (adrenomedullin [ADM] and prostasin [PRSS8]) identified at the first step were associated with chronotype in the fully adjusted model (Figure 3 and Supplementary Table S2). The top five proteins identified with the joint test at both steps were tissue-type plasminogen activator (tPA), retinoic acid receptor responder protein 2 (RARRES2), plasminogen activator inhibitor (PAI-1), fatty-acid binding protein—adipocyte (FABP4), and spondin 2 (SPON2).

**Figure 4.**
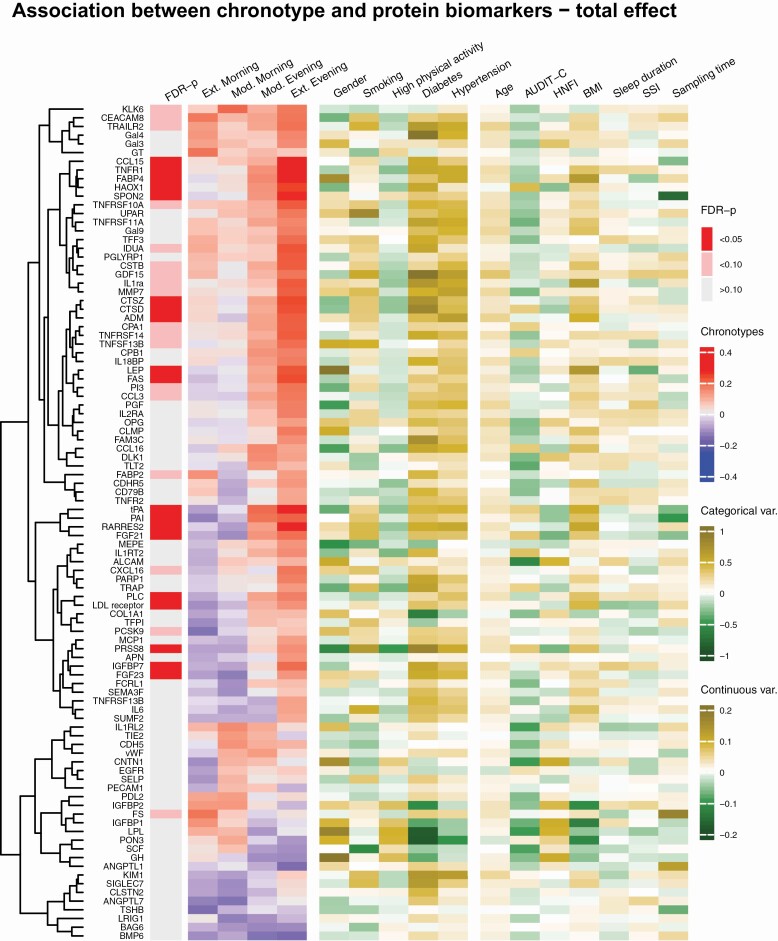
Heatmap on chronotypes, covariates, and clustered protein biomarkers. Figure shows the results for the protein biomarkers associated with chronotype with a Wald-test *p*-value < 0.20. The values for the chronotype categories represent β-coefficients of linear regressions adjusted for age, sex, and sampling time. For the covariates, the values represent β-coefficients of univariate linear regressions. The dendrogram depicts the hierarchical clustering of protein biomarkers based on the Euclidean distance of the regression coefficients for the chronotype categories. The Wald-test was used to jointly test the β-coefficients for all chronotype categories. The intermediate chronotype category was set as the reference. FDR-*p* are the Wald-test *p*-values adjusted for multiple comparison using the Benjamini–Hochberg method. Ext, extreme; Even, evening-type; Mod, moderate; Morn, morning-type; AUDIT-C, Alcohol Use Disorders Identifications Test-Concise score; BMI, body mass index; HNFI, Healthy Nordic Food Index score; SSI, sleep sufficiency index. Full name of the proteins in the heatmap: see Supplementary Table S8.

When examining the effect by each of the chronotype categories, the associations identified were largely driven by positive β-coefficients for the moderate and extreme evening-types. Regression coefficients showed a trend for an association with extreme morning-type for several proteins; however, only PAI-1 was associated after adjustment. For most of the associated proteins, there was a dose-response effect noticed by a progressive increase in the β-coefficients as chronotype delayed in the chronotype scale from the extreme morning-type to the extreme evening-type. This gradient effect could also be observed in a heatmap displaying the association between chronotype categories and the protein biomarkers (Figure 4).

### Effect of insufficient sleep

From the 19 proteins identified at the first step, only RARRES2 was associated with the interaction term of chronotype and insufficient sleep (joint test *p*-value = 0.035) (Table S3).

### Sensitivity analysis

Excluding shift workers from the study sample did not notably change the results (Table S4), nor did adjusting for waist-to-hip ratio and body fat percentage (Table S5).

When we restricted the total effect analysis to the participants who did not have hypertension, diabetes, dyslipidemia, or used medication for these conditions (*n* = 1,459), the direction of the associations was preserved (Table S6). The association with the extreme chronotype was strengthened for some proteins (e.g. tPA, RARRES2, and PAI-1) while attenuated for others. Only four proteins were no longer associated with chronotype (tumor necrosis factor receptor superfamily member 6 [FAS], PRSS8, ADM, and cathepsin Z [CTSZ]).

The complete case analysis and the multiple imputations produced comparable results to the fully adjusted model, with coefficients of similar magnitude and same direction, despite the larger confidence intervals (Table S7). Out of the 17 proteins identified with multiple imputations, four were no longer associated with chronotype in the complete case analysis (fibroblast growth factor 21 [FGF21] and 23 [FGF23], cathepsin D [CTSD], and CTSZ).

## Discussion

The main finding of this study is that chronotype, and especially the evening chronotypes, is associated with circulating cardiometabolic proteins. Out of preselected 242 proteins relevant for CVD and metabolism measured using a high-throughput target proteomics approach, we found 19 proteins associated with chronotype in 2,436 individuals aged 45–75 from Sweden. After further adjustment for hypertension, diabetes, BMI, and other common risk factors of cardiometabolic disorders, 17 proteins remained associated. The associations were mainly driven by a positive association with the evening chronotypes, accompanied by coefficients in the oppositive direction for morning chronotypes.

To our knowledge, this is the first study to investigate the proteomic profile associated with chronotype. Our findings add to the existing knowledge and provide some molecular insights in the relation of chronotype with cardiometabolic disorders. Furthermore, the variety of proteins identified indicates that several mechanisms may link chronotype to health outcomes.

Both PAI-1 and tPA were positively associated with the extreme evening chronotype and PAI-1 was also negatively associated with the extreme morning chronotype even after adjustment for common cardiometabolic risk factors. PAI-1 and tPA have previously been associated with glucose metabolism disorders [29], incident T2D [30], and coronary heart disease [31, 32]. PAI-1 and tPA are components of the fibrinolytic system, with PAI-1 acting as a direct inhibitor of tPA [33]. This is the first time that fibrinolysis markers are linked to chronotype. Prior studies have found that altered fibrinolysis markers can anticipate the development of T2D, even though a causal relationship has not been recognized [34]. In addition, lifestyle interventions for weight reduction through low-fat diet and physical activity led to a reduction in the plasma concentration of these proteins in individuals at high risk for T2D [35, 36].

Lifestyle factors have been suggested as a link between chronotype and cardiometabolic disease. Evening chronotypes have a higher risk of T2D and CVD [5, 6, 37], which could be related to engagement in more unhealthy behaviors [38]. Nevertheless, the particular contribution of each lifestyle factor is unclear. In the current study, among the evening-types, there was a higher proportion of current smokers, lower proportion of individuals with medium-high or high physical activity, but no difference in the diet score. In a UK study (*n* = 635) using wrist accelerometer-based data, evening-types not only were less involved in moderate-to-vigorous physical activity but also spent more time in sedentary behavior [39]. The role of diet as mediator between evening-types and obesity was investigated in the National FINRISK 2007 Study (*n* = 4,421). Although evening-types had lower adherence to a healthy Nordic diet, there was insufficient evidence to conclude that the poorer diet was a mediator between chronotype and obesity. Occupation, especially shift work, could be another factor connecting chronotype to cardiometabolic diseases [11]. Nevertheless, the exclusion of shift workers did not affect our results.

Among our findings, there were three adipokines positively associated with the evening chronotype, namely FABP4, Leptin (LEP), and RARRES2. The intracellular lipid transporter FABP4 is secreted by adipocytes under fasting-related signals and stimulates hepatocyte glucose production [40]. In humans, serum FABP4 has been associated with obesity and insulin resistance [41]. LEP is a well-known appetite regulator produced by the adipose tissue. Obese individuals have higher LEP levels but they are also more resistant to its anorexigenic effect [42]. RARRES-2, also known as chemerin, is involved in adipogenesis, regulation of inflammation and metabolism, and is considered a potential link between obesity and development of insulin resistance [43]. In this study, the three adipokines were associated with chronotype even after adjustment for BMI, waist-to-hip ratio, and body fat percentage, suggesting that obesity may not be the only mechanism connecting these adipokines to chronotype, although we cannot exclude the possibility of insufficient adjustment.

A study by Nowak *et al.* implemented a similar approach to the present study to investigate protein biomarkers associated with insulin resistance [44]. The study used data from two cohorts from Uppsala, Sweden, one as a discovery sample (mean age 70.2) and another as a validation sample (mean age 77.6). Out of the seven proteins identified by Nowak *et al.*, four proteins (LEP, tPA, FABP4, and CTSD) were also identified in the current study, indicating possible pathways linking chronotype and insulin resistance. These findings deserve further exploration in future studies.

For our analysis, we defined the intermediate chronotype as the reference category, and interestingly the resulting regression coefficients for the extreme chronotypes were of opposite directions. Such finding suggests, at least for many associations, the existence of a continuum in protein concentration from the morning-types to the evening-types. Moreover, it supports the validity of our chronotype assessment.

In our study, the main exposure was determined using a single question about preference on time of the day, producing a categorical variable with five levels. Other methods exist, including more detailed questionnaires, as the Morning-Evening Questionnaire [45], and the Munich ChronoType Questionnaire (MCQT) that defines the chronotype on a continuous scale based on the mid-point between bedtime and wakeup-time on free days [46]. Two aspects can underline the reliability of our approach. First, self-assessment of chronotype has been shown to have a high correlation with the MCTQ. Second, the question used in EpiHealth has been previously related to sleep timing and the estimated circadian phase [47]. Although exposure misclassification may not be completely ruled out, it would likely be unrelated to the outcome, resulting in effect estimates biased toward the null.

### Chronotype and insufficient sleep

Given that insufficient sleep has been previously associated with later chronotypes [48] and metabolic disorders [49], we conducted an additional analysis to investigate if the effect of chronotype varied according to the presence or absence of insufficient sleep. Only for one protein, RARRES2, there was a significant interaction for chronotype with insufficient sleep. This result must be interpreted with caution but indicates that the associations found between chronotype and the circulating protein biomarkers are not altered by insufficient sleep.

### Strengths and limitations

The main strengths of this study are the extensively validated proteomics profiling method used [20] and the large and well-characterized study population. The PEA method requires a small sample volume and enabled us to examine 242 circulating proteins biomarkers in relation to chronotype. Because samples were collected over 10 h, the measurements are more likely to reflect average protein levels during the collection period rather than phase shift on plasma levels. Nevertheless, all models were adjusted for sampling time to control for the potential effect of circadian variation on our results. In addition, previous studies have shown that the proteomic profile is usually stable over time [50]. The EpiHealth cohort contains a large number of variables on the participants’ health, lifestyle, and several anthropometric measurements recorded on site [17]. The amount of information available allowed us to condition our analysis for many relevant covariates.

There are, nonetheless, important considerations to be made when assessing the results. Despite the extensive adjustments made, residual confounding is expected to affect the results of observational studies that are so intricately related to lifestyle factors. In addition, most of the data is self-reported, hence susceptible to reporting bias. The cross-sectional design restricts our possibilities of inferring causality, but given the current knowledge, it is unlikely that the protein biomarkers identified could act influencing the chronotype. Although the biomarkers investigated are a preselected set of candidates or established markers for cardiovascular or metabolic disease, many of the markers are involved in multiple biological pathways. Experimental studies are needed before we can draw conclusions about specific pathways. Our findings are also restricted to one study population and we lack a replication cohort. Given the influence of latitude and age on chronotype [51], our findings from a cohort in Uppsala, Sweden (latitude 59°N) need to be validated in other latitudes and other age groups.

Last, other studies have reported a higher social-jetlag, difference in sleep timing between weekdays and weekends [46, 48], in evening-types. Unfortunately, we do not have the data to assess the impact of social-jetlag on our results.

## Conclusion

In this study, we identified several circulating protein biomarkers of cardiometabolic relevance associated with the evening chronotypes. In addition, there was a gradient effect on circulating protein from one extreme to the other in the chronotype scale. Among the newly identified associations, there were proteins previously linked to cardiovascular and metabolic disorders, in particular fibrinolysis markers and adipokines. Overall, these findings suggest potential pathways in the relation of chronotype with health problems, which deserves future investigations.

## Supplementary Material

zsab226_suppl_Supplementary_Materials

## Data Availability

The data underlying this article were provided by the EpiHealth cohort study under the agreement and are not shared publicly due to confidentiality. Data will be shared on request to the corresponding author only after permission of the EpiHealth Steering Committee.
