# Supplementary material for: Evening chronotype is associated with elevated biomarkers of cardiometabolic risk in the EpiHealth cohort: a cross-sectional study
Source: Sleep. 2021 Sep 4;45(2):zsab226. doi: 10.1093/sleep/zsab226 (PMC8842133; doi:10.1093/sleep/zsab226)
Supplement: zsab226_suppl_Supplementary_Materials [file zsab226_suppl_supplementary_materials.docx]

**Title:** Evening chronotype is associated with elevated biomarkers of cardiometabolic risk in the EpiHealth cohort: a cross-sectional study.

**Running head:** Chronotype and protein biomarkers

**Author names and affiliations:**

Gabriel Baldanzi^1^, Ulf Hammar^1^, Tove Fall^1^, Eva Lindberg^2^, Lars Lind^3^, Sölve Elmståhl^4^, Jenny Theorell-Haglöw^1,2^

^1^Department of Medical Sciences, Molecular Epidemiology and Science for Life Laboratory, Uppsala University, 751 85 Uppsala;

^2^Department of Medical Sciences, Respiratory, Allergy and Sleep Research, Uppsala

University, Sweden;

^3^Department of Medical Sciences, Cardiovascular Epidemiology, Uppsala University, Sweden;

^4^ Department of Clinical Sciences in Malmö, Division of Geriatric Medicine, Lund University, Sweden; CRC, Skåne University Hospital, Malmö, Sweden

**Corresponding author's full address and current e-mail:**

Gabriel Baldanzi, Department of Medical Sciences, Molecular Epidemiology Research Group and Science for Life Laboratory, Uppsala University, Uppsala, Sweden;

Epihubben, Dag Hammarskjölds väg 14 B, 75185 Uppsala;

[gabriel.baldanzi@medsci.uu.se](mailto:gabriel.baldanzi@medsci.uu.se)

**Institution where the study was performed:**

Department of Medical Sciences, Molecular Epidemiology Group and the Respiratory, Allergy and Sleep Research Group, Uppsala University, Uppsala, Sweden.

# SUPPLEMENTARY FIGURE AND TABLES

**Figure S1.** Directed acyclic graph on the effect of chronotype on circulating proteins. According to this hypothetical causal network, the total effect of chronotype on protein level can be estimated by adjusting for age and sex. The direct effect of chronotype can be estimated by adjusting for age, sex, body mass index (BMI), sleep duration, sleep sufficiency, diet, physical activity, alcohol, smoking, type 2 diabetes (T2D), and hypertension.

**Table S1.** Association between chronotype and circulating protein biomarkers (Step 1. Total effect; N=2,436). Associations between chronotype and circulating protein biomarkers using linear regression models with each protein as the outcome, adjusted for age, sex, sampling time and multiple testing (FDR 5%). Results are presented as β-coefficients, 95% confidence intervals, and p-values, and show changes in protein measurement in relation to the intermediate chronotype.

**Table S2.** Association between chronotype and circulating protein biomarkers (Step 2. Direct effect; N=2,436). Associations between chronotype and circulating proteins biomarkers using linear models with each protein as the outcome, adjusted for age, sex, sampling time, smoking, alcohol, physical activity, diet, sleep sufficiency, sleep duration, BMI, hypertension, and diabetes. Proteins shown were significant at the 5% FDR at step 1. Results are presented as β-coefficients, 95% confidence intervals, and p-values, and show changes in protein measurement in relation to the intermediate chronotype.

**Table S3**. Interaction between chronotype and insufficient sleep. Associations between chronotype and circulating protein biomarkers using linear regression models with each protein as the outcome, adjusted for insufficient sleep, age, sex, sampling time, and an interaction term of chronotype with insufficient sleep. Results are presented as the Wald-test p-value comparing the interaction term coefficients of each chronotype categories for each protein.

## Table S4. Association between chronotype and circulating protein biomarkers in non-shift workers (N=2,312). Associations between chronotype and circulating proteins biomarkers in non-shift workers using linear model with each protein as the outcome, adjusted for age, sex, sampling time, and multiple testing (FDR 5%). Results are presented as β-coefficients, 95% confidence intervals, and p-values, and show protein changes in relation to the intermediate chronotype.

**Table S5.** Association between chronotype and protein biomarkers with additional adjustment for body fat mass (N=2,436). Associations between chronotype and circulating proteins biomarkers using linear regression models with each protein as the outcome, adjusted for age, sex, sampling time, smoking, alcohol, physical activity, diet, sleep sufficiency, sleep duration, BMI, hypertension, diabetes, body fat percentage, and waist-to-hip ratio. Proteins shown were significant at the 5% FDR at step 1. Results are presented as β-coefficients, 95% confidence intervals, and p-values, and show changes in protein measurement in relation to the intermediate chronotype.

**Table S6.** Association between chronotype and circulating protein biomarkers among participants without hypertension, diabetes, dyslipidemia, or medication use for these conditions (N=1,459). Each of 19 proteins associated with chronotype in the first step was modelled as the outcome in a linear regression adjusted for age, sex, and sampling time. Results are presented as β-coefficients, 95% confidence intervals, and p-values, and show changes in protein measurement in relation to the intermediate chronotype.

**Table S7.** Association between chronotype and circulating protein biomarkers from complete case analysis. Associations between chronotype and circulating proteins biomarkers using linear models with each protein as the outcome, adjusted for age, sex, sampling time, smoking, alcohol, physical activity, diet, sleep sufficiency, sleep duration, BMI, hypertension, and diabetes. Proteins shown were previously associated with chronotype at the step 2 using the imputed data. Results are presented as β-coefficients, 95% confidence intervals, and p-values, and show changes in protein measurement in relation to the intermediate chronotype.

**Table S8.** Full name of proteins added to the heatmap (Fig. 4). These proteins were associated with chronotype with a Wald-test p-value<0.20 in linear regression models adjusted for age, sex, and sampling time.

**Figure S1. Directed acyclic graph on the effect of chronotype on circulating proteins.**


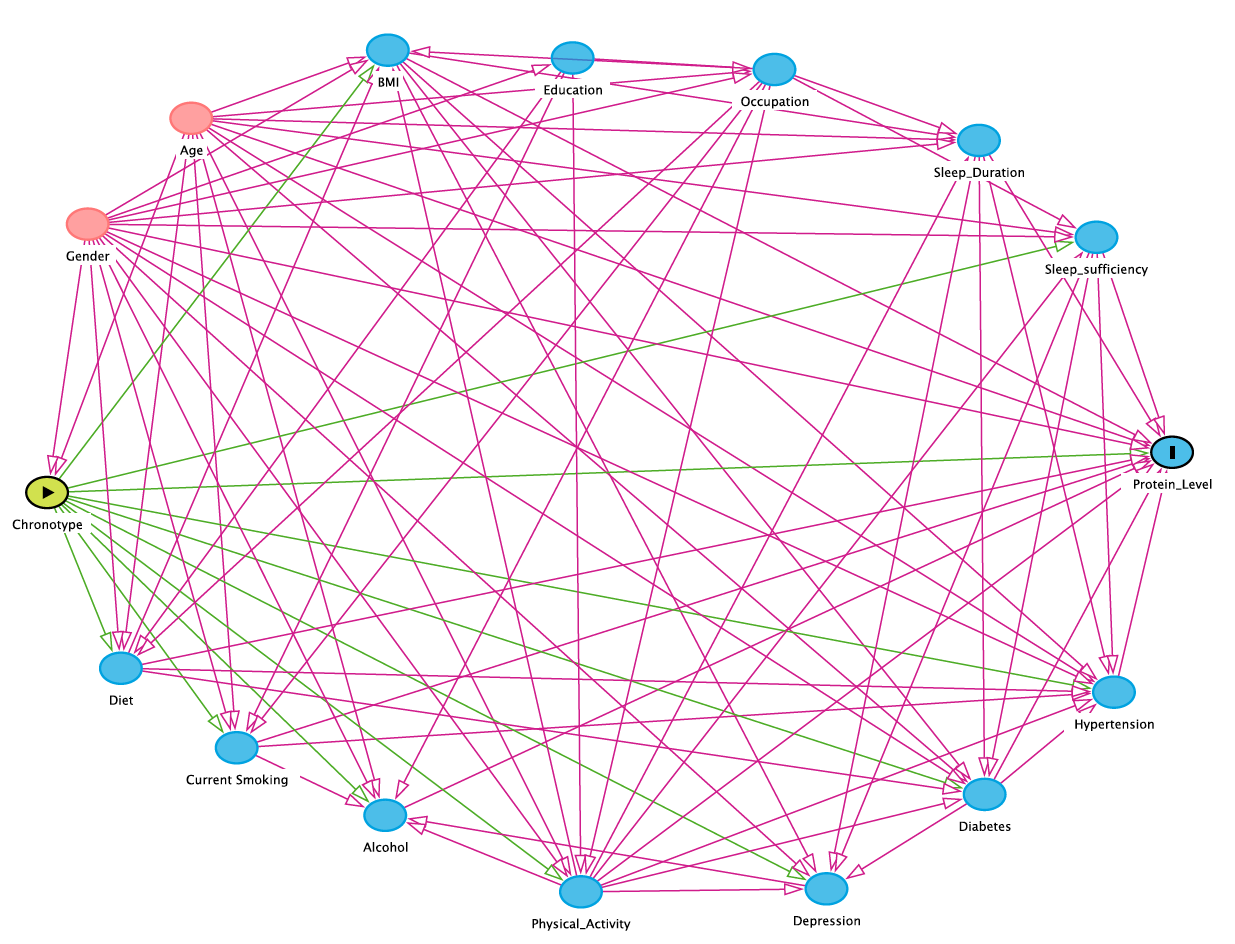


## Table S1. Association between chronotype and circulating protein biomarkers (Step 1. Total effect; N=2,436)

|  | | | Extreme morning | | Moderately morning | | Moderately evening | | Extreme evening | |
| --- | --- | --- | --- | --- | --- | --- | --- | --- | --- | --- |
| Protein | Wald-test p | FDR-p | β | p-value | β | p-value | β | p-value | β | p-value |
| tPA | **6.55e-11** | **1.59e-08** | -0.10 [-0.20, 0.01] | 0.075 | -0.01 [-0.13, 0.10] | 0.824 | 0.19 [0.07, 0.31] | **2.62e-03** | 0.29 [0.18, 0.39] | **1.46e-07** |
| RARRES2 | **6.45e-08** | **7.81e-06** | -0.03 [-0.14, 0.08] | 0.580 | -0.10 [-0.22, 0.02] | **0.111** | 0.11 [-0.01, 0.24] | 0.081 | 0.27 [0.16, 0.38] | **2.16e-06** |
| PAI-1 | **1.67e-07** | **1.34e-05** | -0.13 [-0.24, -0.03] | **0.013** | -0.06 [-0.17, 0.06] | 0.332 | 0.18 [0.05, 0.31] | **0.007** | 0.20 [0.08, 0.32] | **7.66e-04** |
| FABP4 | **2.43e-06** | **1.47e-04** | -0.02 [-0.12, 0.08] | 0.670 | 0.02 [-0.08, 0.13] | 0.654 | 0.15 [0.02, 0.27] | **0.019** | 0.26 [0.16, 0.37] | **1.17e-06** |
| SPON2 | **8.00e-06** | **3.87e-04** | -0.03 [-0.13, 0.07] | 0.579 | -0.01 [-0.11, 0.10] | 0.893 | 0.13 [0.01, 0.25] | **0.040** | 0.26 [0.15, 0.38] | **5.90e-06** |
| LEP | **3.75e-05** | **1.44e-03** | -0.03 [-0.12, 0.06] | 0.543 | -0.00 [-0.11, 0.10] | 0.929 | 0.10 [-0.01, 0.21] | 0.073 | 0.22 [0.12, 0.32] | **2.43e-05** |
| TNFR1 | **4.20e-05** | **1.44e-03** | 0.02 [-0.09, 0.12] | 0.782 | 0.01 [-0.11, 0.12] | 0.887 | 0.14 [0.01, 0.26] | **0.031** | 0.27 [0.15, 0.38] | **4.72e-06** |
| FGF21 | **4.76e-05** | **1.44e-03** | -0.07 [-0.18, 0.04] | 0.186 | -0.12 [-0.24, 0.00] | 0.053 | 0.13 [-0.00, 0.26] | 0.050 | 0.18 [0.06, 0.30] | **2.51e-03** |
| CCL15 | **1.29e-04** | **3.46e-03** | 0.02 [-0.09, 0.13] | 0.702 | 0.06 [-0.06, 0.18] | 0.344 | 0.09 [-0.03, 0.21] | 0.146 | 0.29 [0.17, 0.41] | **3.75e-06** |
| FAS | **4.84e-04** | **0.012** | -0.06 [-0.16, 0.05] | 0.313 | -0.01 [-0.12, 0.10] | 0.798 | 0.11 [-0.03, 0.25] | 0.120 | 0.23 [0.10, 0.36] | **3.75e-04** |
| ADM | **7.94e-04** | **0.017** | 0.03 [-0.07, 0.14] | 0.531 | -0.05 [-0.15, 0.06] | 0.368 | 0.11 [-0.01, 0.23] | 0.084 | 0.21 [0.09, 0.32] | **3.74e-04** |
| LDL receptor | **1.78e-03** | **0.034** | -0.11 [-0.22, -0.00] | 0.048 | -0.06 [-0.19, 0.06] | 0.328 | 0.09 [-0.04, 0.21] | 0.194 | 0.14 [0.01, 0.26] | **0.033** |
| CTSD | **1.93e-03** | **0.034** | 0.03 [-0.08, 0.14] | 0.577 | -0.01 [-0.13, 0.11] | 0.900 | 0.10 [-0.03, 0.23] | 0.127 | 0.22 [0.11, 0.34] | **1.79e-04** |
| PLC | **1.96e-03** | **0.034** | -0.08 [-0.22, 0.05] | 0.219 | -0.02 [-0.13, 0.08] | 0.657 | 0.11 [-0.00, 0.22] | 0.055 | 0.16 [0.05, 0.27] | **3.74e-03** |
| CTSZ | **2.09e-03** | **0.034** | 0.04 [-0.07, 0.14] | 0.523 | -0.02 [-0.13, 0.10] | 0.766 | 0.11 [-0.02, 0.24] | 0.092 | 0.23 [0.11, 0.36] | **2.25e-04** |
| IGFBP7 | **2.26e-03** | **0.034** | -0.07 [-0.18, 0.04] | 0.195 | -0.09 [-0.21, 0.03] | 0.140 | 0.02 [-0.10, 0.14] | 0.717 | 0.19 [0.06, 0.32] | **4.64e-03** |
| HAOX1 | **2.44e-03** | **0.034** | -0.00 [-0.11, 0.10] | 0.966 | 0.02 [-0.10, 0.14] | 0.733 | 0.13 [0.00, 0.26] | **0.045** | 0.24 [0.11, 0.37] | **4.28e-04** |
| FGF23 | **2.51e-03** | **0.034** | -0.10 [-0.21, 0.01] | 0.088 | -0.14 [-0.26, -0.03] | **0.015** | -0.00 [-0.14, 0.13] | 0.947 | 0.13 [-0.00, 0.25] | 0.051 |
| PRSS8 | **2.82e-03** | **0.036** | -0.09 [-0.19, 0.01] | 0.079 | -0.09 [-0.20, 0.02] | 0.114 | 0.04 [-0.08, 0.17] | 0.502 | 0.14 [0.02, 0.26] | **0.023** |

ADM: Adrenomedullin, CCL15: C-C motif chemokine 15, CTSD: cathepsin D, CTSZ: cathepsin Z, FAS: tumor necrosis factor receptor superfamily member 6, FABP4: fatty acid-binding protein, adipocyte, FGF21: fibroblast growth factor 21, FGF23: fibroblast growth factor 23, HAOX1: hydroxyacid oxidase 1, IGFBP7: insulin-like growth factor-binding protein 7, LEP: leptin, LDLreceptor: low-density lipoprotein receptor, PAI-1: plasminogen activator inhibitor 1, PLC: perlecan, PRSS8: prostasin, RARRES2: retinoic acid receptor protein 2, SPON2: spondin-2, tPA: tissue-type plasminogen activator. TNFR1: tumor necrosis factor receptor 1.

## Table S2. Association between chronotype and circulating protein biomarkers (Step 2. Direct effect; N=2,436)

|  | | Extreme morning | | Moderately morning | | Moderately evening | | Extreme evening | |
| --- | --- | --- | --- | --- | --- | --- | --- | --- | --- |
| Protein | Wald-test p | β | p-value | β | p-value | β | p-value | β | p-value |
| tPA | **1.45e-07** | -0.09 [-0.18, 0.01] | 0.079 | 0.01 [-0.10, 0.12] | 0.841 | 0.15 [0.03, 0.26] | **0.011** | 0.22 [0.12, 0.32] | **1.60e-05** |
| SPON2 | **1.70e-05** | -0.04 [-0.14, 0.06] | 0.454 | -0.00 [-0.11, 0.10] | 0.941 | 0.13 [0.01, 0.25] | **0.033** | 0.24 [0.13, 0.36] | **2.06e-05** |
| PAI-1 | **1.72e-05** | -0.13 [-0.23, -0.03] | **0.013** | -0.04 [-0.14, 0.07] | 0.531 | 0.15 [0.03, 0.27] | **0.018** | 0.14 [0.03, 0.25] | **0.011** |
| RARRES2 | **1.19e-04** | -0.03 [-0.13, 0.07] | 0.570 | -0.07 [-0.18, 0.03] | 0.184 | 0.08 [-0.04, 0.19] | 0.176 | 0.18 [0.08, 0.29] | **3.34e-04** |
| FABP4 | **1.23e-04** | -0.01 [-0.09, 0.07] | 0.827 | 0.05 [-0.03, 0.14] | 0.198 | 0.11 [0.01, 0.21] | **0.026** | 0.18 [0.10, 0.27] | **1.85e-05** |
| TNFR1 | **4.40e-04** | 0.00 [-0.10, 0.11] | 0.929 | 0.02 [-0.10, 0.13] | 0.761 | 0.13 [0.01, 0.25] | **0.028** | 0.23 [0.12, 0.34] | **5.94e-05** |
| LEP | **6.82e-04** | -0.01 [-0.08, 0.06] | 0.747 | 0.03 [-0.04, 0.10] | 0.398 | 0.06 [-0.02, 0.14] | 0.168 | 0.14 [0.07, 0.21] | **1.12e-04** |
| CCL15 | **8.77e-04** | 0.02 [-0.09, 0.13] | 0.745 | 0.06 [-0.06, 0.18] | 0.306 | 0.10 [-0.03, 0.22] | 0.123 | 0.26 [0.14, 0.38] | **3.75e-05** |
| FAS | **1.30e-03** | -0.07 [-0.17, 0.04] | 0.223 | -0.01 [-0.12, 0.10] | 0.876 | 0.11 [-0.03, 0.24] | 0.129 | 0.20 [0.08, 0.33] | **1.31e-03** |
| PLC | **4.14e-03** | -0.08 [-0.21, 0.04] | 0.200 | -0.02 [-0.12, 0.09] | 0.745 | 0.10 [-0.00, 0.21] | 0.056 | 0.14 [0.04, 0.24] | **7.61e-03** |
| FGF21 | **5.66e-03** | -0.07 [-0.17, 0.04] | 0.208 | -0.10 [-0.21, 0.02] | 0.096 | 0.09 [-0.03, 0.21] | 0.130 | 0.12 [0.00, 0.23] | **0.047** |
| IGFBP7 | **6.23e-03** | -0.07 [-0.18, 0.04] | 0.231 | -0.09 [-0.20, 0.03] | 0.155 | 0.03 [-0.09, 0.15] | 0.590 | 0.17 [0.04, 0.30] | **0.010** |
| FGF23 | **0.010** | -0.11 [-0.21, 0.00] | 0.055 | -0.13 [-0.25, -0.02] | **0.018** | -0.00 [-0.13, 0.13] | 0.976 | 0.08 [-0.04, 0.21] | 0.187 |
| HAOX1 | **0.013** | 0.02 [-0.09, 0.12] | 0.756 | 0.04 [-0.07, 0.15] | 0.498 | 0.12 [-0.00, 0.25] | 0.058 | 0.22 [0.09, 0.35] | **1.21e-03** |
| LDL receptor | **0.024** | -0.10 [-0.21, 0.00] | 0.056 | -0.04 [-0.16, 0.08] | 0.511 | 0.06 [-0.06, 0.19] | 0.340 | 0.09 [-0.03, 0.21] | 0.122 |
| CTSZ | **0.024** | 0.03 [-0.07, 0.14] | 0.550 | -0.00 [-0.11, 0.11] | 0.962 | 0.10 [-0.02, 0.23] | 0.100 | 0.18 [0.06, 0.30] | **2.47e-03** |
| CTSD | **0.034** | 0.04 [-0.06, 0.15] | 0.451 | 0.01 [-0.10, 0.13] | 0.820 | 0.09 [-0.03, 0.21] | 0.153 | 0.17 [0.06, 0.28] | **2.44e-03** |
| ADM | 0.053 | 0.04 [-0.06, 0.13] | 0.465 | -0.03 [-0.12, 0.07] | 0.589 | 0.07 [-0.04, 0.19] | 0.186 | 0.13 [0.03, 0.24] | **0.011** |
| PRSS8 | 0.091 | -0.09 [-0.18, 0.01] | 0.064 | -0.07 [-0.17, 0.04] | 0.218 | 0.02 [-0.10, 0.14] | 0.756 | 0.06 [-0.05, 0.17] | 0.314 |

ADM: Adrenomedullin, CCL15: C-C motif chemokine 15, CTSD: cathepsin D, CTSZ: cathepsin Z, FAS: tumor necrosis factor receptor superfamily member 6, FABP4: fatty acid-binding protein, adipocyte, FGF21: fibroblast growth factor 21, FGF23: fibroblast growth factor 23, HAOX1: hydroxyacid oxidase 1, IGFBP7: insulin-like growth factor-binding protein 7, LEP: leptin, LDLreceptor: low-density lipoprotein receptor, PAI-1: plasminogen activator inhibitor 1, PLC: perlecan, PRSS8: prostasin, RARRES2: retinoic acid receptor protein 2, SPON2: spondin-2, tPA: tissue-type plasminogen activator. TNFR1: tumor necrosis factor receptor 1.

## Table S3.

**Interaction between chronotype and insufficient sleep**

| Protein | Wald-test p-value for the interaction term |
| --- | --- |
| RARRES2 | 0.035 |
| CTSD | 0.139 |
| FABP4 | 0.176 |
| LEP | 0.207 |
| tPA | 0.214 |
| FGF23 | 0.223 |
| PLC | 0.226 |
| LDL receptor | 0.252 |
| ADM | 0.495 |
| TNFR1 | 0.550 |
| HAOX1 | 0.653 |
| PAI-1 | 0.720 |
| PRSS8 | 0.764 |
| IGFBP7 | 0.784 |
| CTSZ | 0.799 |
| CCL15 | 0.842 |
| FGF21 | 0.867 |
| FAS | 0.871 |
| SPON2 | 0.926 |

ADM: Adrenomedullin, CCL15: C-C motif chemokine 15, CTSD: cathepsin D, CTSZ: cathepsin Z, FAS: tumor necrosis factor receptor superfamily member 6, FABP4: fatty acid-binding protein, adipocyte, FGF21: fibroblast growth factor 21, FGF23: fibroblast growth factor 23, HAOX1: hydroxyacid oxidase 1, IGFBP7: insulin-like growth factor-binding protein 7, LEP: leptin, LDLreceptor: low-density lipoprotein receptor, PAI-1: plasminogen activator inhibitor 1, PLC: perlecan, PRSS8: prostasin, RARRES2: retinoic acid receptor protein 2, SPON2: spondin-2, tPA: tissue-type plasminogen activator. TNFR1: tumor necrosis factor receptor 1.

## Table S4. Association between chronotype and circulating protein biomarkers in non-shift workers (N=2,312)

|  | | | Extreme morning | | Moderately morning | | Moderately evening | | Extreme evening | |
| --- | --- | --- | --- | --- | --- | --- | --- | --- | --- | --- |
| Protein | Wald-test p | FDR-p | β | p-value | β | p-value | β | p-value | β | p-value |
| tPA | **1.58e-09** | **3.82e-07** | -0.07 [-0.18, 0.04] | 0.192 | -0.02 [-0.14, 0.10] | 0.768 | 0.21 [0.08, 0.34] | **1.03e-03** | 0.27 [0.17, 0.38] | **9.45e-07** |
| RARRES2 | **1.79e-07** | **2.17e-05** | -0.02 [-0.14, 0.09] | 0.703 | -0.11 [-0.23, 0.01] | 0.082 | 0.14 [0.01, 0.27] | **0.039** | 0.26 [0.15, 0.37] | **7.61e-06** |
| PAI-1 | **3.64e-07** | **2.94e-05** | -0.12 [-0.23, -0.02] | 0.025 | -0.05 [-0.16, 0.07] | 0.447 | 0.20 [0.06, 0.33] | **3.71e-03** | 0.20 [0.08, 0.32] | **8.20e-04** |
| FABP4 | **4.71e-06** | **2.85e-04** | -0.01 [-0.12, 0.09] | 0.796 | 0.03 [-0.08, 0.13] | 0.613 | 0.16 [0.04, 0.29] | **0.012** | 0.27 [0.16, 0.38] | **1.76e-06** |
| SPON2 | **3.64e-05** | **1.76e-03** | -0.03 [-0.13, 0.07] | 0.601 | -0.01 [-0.12, 0.09] | 0.809 | 0.14 [0.02, 0.27] | **0.022** | 0.25 [0.13, 0.36] | **3.69e-05** |
| TNFR1 | **6.56e-05** | **2.64e-03** | 0.02 [-0.09, 0.13] | 0.707 | 0.01 [-0.11, 0.13] | 0.869 | 0.16 [0.04, 0.29] | **0.012** | 0.27 [0.15, 0.38] | **1.18e-05** |
| LEP | **9.78e-05** | **3.38e-03** | -0.02 [-0.11, 0.08] | 0.721 | -0.01 [-0.11, 0.09] | 0.857 | 0.10 [-0.01, 0.22] | 0.074 | 0.22 [0.12, 0.33] | **3.83e-05** |
| FGF21 | **2.61e-04** | **7.70e-03** | -0.05 [-0.16, 0.06] | 0.362 | -0.13 [-0.25, -0.00] | 0.042 | 0.14 [0.01, 0.28] | **0.042** | 0.16 [0.04, 0.28] | **9.29e-03** |
| CCL15 | **2.86e-04** | **7.70e-03** | 0.02 [-0.10, 0.13] | 0.787 | 0.07 [-0.06, 0.19] | 0.295 | 0.11 [-0.02, 0.23] | 0.091 | 0.28 [0.16, 0.41] | **1.12e-05** |
| ADM | **1.19e-03** | **0.029** | 0.05 [-0.06, 0.16] | 0.380 | -0.04 [-0.15, 0.07] | 0.449 | 0.13 [0.01, 0.26] | **0.041** | 0.20 [0.09, 0.32] | **6.63e-04** |
| FAS | **1.90e-03** | **0.042** | -0.06 [-0.17, 0.05] | 0.312 | -0.00 [-0.11, 0.11] | 0.998 | 0.14 [-0.01, 0.28] | 0.061 | 0.19 [0.07, 0.31] | **2.34e-03** |
| LDL receptor | **2.17e-03** | **0.044** | -0.10 [-0.21, 0.02] | 0.094 | -0.07 [-0.19, 0.06] | 0.298 | 0.10 [-0.03, 0.24] | **0.122** | 0.14 [0.02, 0.27] | **2.69e-02** |

ADM: Adrenomedullin, CCL15: C-C motif chemokine 15, FAS: tumor necrosis factor receptor superfamily member 6, FABP4: fatty acid-binding protein, adipocyte, FGF21: fibroblast growth factor 21, LEP: leptin, LDL receptor: low-density lipoprotein receptor, PAI-1: plasminogen activator inhibitor 1, RARRES2: retinoic acid receptor protein 2, SPON2: spondin-2, tPA: tissue-type plasminogen activator, TNFR1: tumor necrosis factor receptor 1.

## Table S5. Association between chronotype and protein biomarkers with additional adjustment for body fat mass (N=2,436)

|  | | Extreme morning | | Moderately morning | | Moderately evening | | Extreme evening | |
| --- | --- | --- | --- | --- | --- | --- | --- | --- | --- |
| Protein | Wald-test p | β | p-value | β | p-value | β | p-value | β | p-value |
| tPA | **1.84e-07** | -0.09 [-0.19, 0.00] | 0.058 | -0.00 [-0.11, 0.10] | 0.971 | 0.14 [0.03, 0.25] | **0.012** | 0.21 [0.11, 0.31] | **3.08e-05** |
| PAI-1 | **1.48e-05** | -0.13 [-0.23, -0.03] | **0.010** | -0.05 [-0.15, 0.06] | 0.416 | 0.15 [0.03, 0.27] | **0.017** | 0.13 [0.02, 0.24] | **0.018** |
| SPON2 | **1.65e-05** | -0.04 [-0.13, 0.06] | 0.459 | -0.00 [-0.11, 0.10] | 0.950 | 0.13 [0.01, 0.25] | **0.034** | 0.25 [0.13, 0.36] | **1.95e-05** |
| RARRES2 | **8.05e-05** | -0.04 [-0.13, 0.06] | 0.468 | -0.09 [-0.19, 0.02] | 0.095 | 0.07 [-0.04, 0.18] | 0.210 | 0.17 [0.08, 0.27] | **5.17e-04** |
| FABP4 | **9.71e-05** | -0.01 [-0.09, 0.06] | 0.742 | 0.04 [-0.04, 0.13] | 0.285 | 0.10 [0.01, 0.20] | **0.031** | 0.18 [0.10, 0.26] | **1.50e-05** |
| LEP | **1.21e-04** | -0.02 [-0.08, 0.04] | 0.519 | 0.01 [-0.05, 0.07] | 0.705 | 0.04 [-0.03, 0.11] | 0.226 | 0.13 [0.07, 0.20] | **4.05e-05** |
| TNFR1 | **4.67e-04** | 0.00 [-0.10, 0.11] | 0.937 | 0.02 [-0.10, 0.13] | 0.774 | 0.13 [0.01, 0.25] | **0.029** | 0.22 [0.11, 0.33] | **6.58e-05** |
| CCL15 | **9.62e-04** | 0.02 [-0.09, 0.13] | 0.762 | 0.06 [-0.06, 0.18] | 0.326 | 0.09 [-0.03, 0.22] | 0.129 | 0.26 [0.13, 0.38] | **4.23e-05** |
| FAS | **1.38e-03** | -0.07 [-0.18, 0.04] | 0.216 | -0.01 [-0.12, 0.10] | 0.856 | 0.11 [-0.03, 0.24] | 0.126 | 0.20 [0.08, 0.33] | **1.50e-03** |
| FGF21 | **3.86e-03** | -0.07 [-0.18, 0.03] | 0.161 | -0.11 [-0.22, -0.00] | **0.049** | 0.09 [-0.03, 0.21] | 0.138 | 0.10 [-0.01, 0.22] | 0.075 |
| PLC | **4.05e-03** | -0.08 [-0.21, 0.04] | 0.201 | -0.02 [-0.12, 0.09] | 0.739 | 0.10 [-0.00, 0.21] | 0.062 | 0.14 [0.04, 0.24] | **7.02e-03** |
| IGFBP7 | **6.08e-03** | -0.07 [-0.18, 0.04] | 0.245 | -0.08 [-0.20, 0.04] | 0.176 | 0.03 [-0.08, 0.15] | 0.570 | 0.17 [0.05, 0.30] | **7.82e-03** |
| FGF23 | **9.47e-03** | -0.11 [-0.21, 0.00] | 0.053 | -0.14 [-0.25, -0.03] | **0.016** | -0.00 [-0.13, 0.13] | 0.973 | 0.08 [-0.04, 0.20] | 0.197 |
| HAOX1 | **0.016** | 0.01 [-0.09, 0.12] | 0.828 | 0.03 [-0.08, 0.14] | 0.621 | 0.12 [-0.01, 0.24] | 0.067 | 0.21 [0.08, 0.34] | **1.80e-03** |
| LDLreceptor | **0.023** | -0.11 [-0.21, -0.00] | **0.041** | -0.05 [-0.17, 0.06] | 0.381 | 0.06 [-0.07, 0.18] | 0.372 | 0.08 [-0.04, 0.20] | 0.167 |
| CTSZ | **0.028** | 0.03 [-0.08, 0.13] | 0.596 | -0.01 [-0.12, 0.10] | 0.862 | 0.10 [-0.02, 0.22] | 0.101 | 0.18 [0.06, 0.29] | **3.64e-03** |
| CTSD | **0.044** | 0.04 [-0.07, 0.14] | 0.507 | 0.00 [-0.11, 0.12] | 0.960 | 0.09 [-0.03, 0.21] | 0.163 | 0.16 [0.05, 0.28] | **3.95e-03** |
| ADM | 0.052 | 0.03 [-0.06, 0.13] | 0.508 | -0.04 [-0.13, 0.06] | 0.472 | 0.07 [-0.04, 0.18] | 0.208 | 0.13 [0.03, 0.23] | **0.014** |
| PRSS8 | 0.077 | -0.09 [-0.19, -0.00] | **0.048** | -0.08 [-0.18, 0.03] | 0.149 | 0.02 [-0.10, 0.13] | 0.770 | 0.05 [-0.06, 0.16] | 0.406 |

ADM: Adrenomedullin, CCL15: C-C motif chemokine 15, CTSD: cathepsin D, CTSZ: cathepsin Z, FAS: tumor necrosis factor receptor superfamily member 6, FABP4: fatty acid-binding protein, adipocyte, FGF21: fibroblast growth factor 21, FGF23: fibroblast growth factor 23, HAOX1: hydroxyacid oxidase 1, IGFBP7: insulin-like growth factor-binding protein 7, LEP: leptin, LDL receptor: low-density lipoprotein receptor, PAI-1: plasminogen activator inhibitor 1, PLC: perlecan, PRSS8: prostasin, RARRES2: retinoic acid receptor protein 2, SPON2: spondin-2, tPA: tissue-type plasminogen activator. TNFR1: tumor necrosis factor receptor 1.

## Table S6. Association between chronotype and circulating protein biomarkers among participants without hypertension, diabetes, dyslipidemia, or medication use for these conditions (N=1,459)

|  | | Extreme morning | | Moderately morning | | Moderately evening | | Extreme evening | |
| --- | --- | --- | --- | --- | --- | --- | --- | --- | --- |
| Protein | Wald-test p | β | p-value | β | p-value | β | p-value | β | p-value |
| tPA | **1.05e-08** | -0.05 [-0.18, 0.08] | 0.460 | 0.02 [-0.12, 0.17] | 0.746 | 0.27 [0.12, 0.42] | **4.43e-04** | 0.36 [0.22, 0.49] | **3.36e-07** |
| RARRES2 | **2.96e-06** | -0.02 [-0.17, 0.13] | 0.787 | -0.11 [-0.26, 0.05] | 0.174 | 0.16 [0.01, 0.32] | **0.042** | 0.31 [0.16, 0.45] | **2.49e-05** |
| PAI-1 | **1.44e-05** | -0.11 [-0.24, 0.03] | 0.126 | -0.02 [-0.16, 0.13] | 0.835 | 0.20 [0.04, 0.36] | **0.014** | 0.26 [0.12, 0.41] | **3.33e-04** |
| CTSD | **8.16e-05** | 0.03 [-0.11, 0.18] | 0.635 | 0.01 [-0.15, 0.17] | 0.945 | 0.10 [-0.06, 0.27] | 0.208 | 0.34 [0.19, 0.48] | **6.41e-06** |
| FABP4 | **0.001** | 0.01 [-0.11, 0.13] | 0.891 | 0.08 [-0.05, 0.20] | 0.251 | 0.15 [0.01, 0.30] | **0.038** | 0.27 [0.14, 0.40] | **8.84e-05** |
| TNFR1 | **0.002** | -0.00 [-0.13, 0.12] | 0.941 | -0.00 [-0.14, 0.13] | 0.957 | 0.15 [0.01, 0.29] | **0.037** | 0.26 [0.11, 0.40] | **4.63e-04** |
| HAOX1 | **0.004** | 0.04 [-0.10, 0.18] | 0.582 | 0.06 [-0.09, 0.22] | 0.426 | 0.19 [0.03, 0.35] | **0.018** | 0.31 [0.13, 0.48] | **0.001** |
| LEP | **0.007** | 0.02 [-0.11, 0.14] | 0.804 | 0.04 [-0.09, 0.18] | 0.536 | 0.12 [-0.01, 0.26] | 0.070 | 0.24 [0.10, 0.38] | **0.001** |
| FGF21 | **0.009** | -0.01 [-0.15, 0.13] | 0.878 | -0.01 [-0.16, 0.14] | 0.900 | 0.15 [-0.01, 0.30] | 0.075 | 0.25 [0.09, 0.41] | **0.002** |
| CCL15 | **0.024** | -0.04 [-0.19, 0.10] | 0.539 | -0.01 [-0.17, 0.16] | 0.951 | 0.02 [-0.13, 0.17] | 0.765 | 0.23 [0.07, 0.39] | **0.005** |
| SPON2 | **0.025** | -0.02 [-0.15, 0.10] | 0.689 | 0.00 [-0.12, 0.13] | 0.955 | 0.08 [-0.06, 0.23] | 0.260 | 0.22 [0.07, 0.37] | **0.004** |
| FGF23 | **0.029** | -0.14 [-0.27, -0.01] | 0.038 | -0.17 [-0.31, -0.03] | **0.018** | -0.02 [-0.18, 0.14] | 0.828 | 0.05 [-0.10, 0.19] | 0.543 |
| PLC | **0.032** | -0.05 [-0.17, 0.07] | 0.401 | -0.01 [-0.14, 0.12] | 0.850 | 0.10 [-0.04, 0.23] | 0.156 | 0.16 [0.02, 0.29] | **0.020** |
| IGFBP7 | **0.042** | -0.14 [-0.29, -0.00] | 0.043 | -0.11 [-0.26, 0.04] | 0.157 | -0.02 [-0.16, 0.12] | 0.777 | 0.12 [-0.05, 0.28] | 0.175 |
| LDLreceptor | **0.045** | -0.07 [-0.21, 0.07] | 0.333 | 0.00 [-0.16, 0.16] | 0.999 | 0.13 [-0.03, 0.29] | 0.105 | 0.15 [-0.00, 0.31] | **0.050** |
| FAS | 0.182 | -0.04 [-0.20, 0.11] | 0.576 | -0.02 [-0.17, 0.13] | 0.772 | 0.13 [-0.06, 0.32] | 0.169 | 0.13 [-0.03, 0.29] | 0.110 |
| PRSS8 | 0.186 | -0.08 [-0.21, 0.04] | 0.178 | -0.01 [-0.14, 0.13] | 0.930 | 0.04 [-0.10, 0.19] | 0.563 | 0.13 [-0.04, 0.29] | 0.131 |
| ADM | 0.230 | 0.05 [-0.08, 0.18] | 0.447 | 0.02 [-0.10, 0.14] | 0.762 | 0.07 [-0.07, 0.21] | 0.326 | 0.17 [0.02, 0.31] | **0.022** |
| CTSZ | 0.295 | 0.03 [-0.11, 0.17] | 0.681 | -0.01 [-0.15, 0.13] | 0.910 | 0.10 [-0.05, 0.26] | 0.198 | 0.15 [-0.01, 0.31] | 0.063 |

ADM: Adrenomedullin, CCL15: C-C motif chemokine 15, CTSD: cathepsin D, CTSZ: cathepsin Z, FAS: tumor necrosis factor receptor superfamily member 6, FABP4: fatty acid-binding protein, adipocyte, FGF21: fibroblast growth factor 21, FGF23: fibroblast growth factor 23, HAOX1: hydroxyacid oxidase 1, IGFBP7: insulin-like growth factor-binding protein 7, LEP: leptin, LDLreceptor: low-density lipoprotein receptor, PAI-1: plasminogen activator inhibitor 1, PLC: perlecan, PRSS8: prostasin, RARRES2: retinoic acid receptor protein 2, SPON2: spondin-2, tPA: tissue-type plasminogen activator. TNFR1: tumor necrosis factor receptor 1.

**Table S7. Association between chronotype and circulating protein biomarkers (Complete case analysis).**

|  | | Extreme morning | | Moderately morning | | Moderately evening | | Extreme evening | |  |
| --- | --- | --- | --- | --- | --- | --- | --- | --- | --- | --- |
| Protein | Wald-test p | β | p-value | β | p-value | β | p-value | β | p-value | N |
| tPA | **2.86e-06** | -0.09 [-0.20, 0.03] | 0.134 | -0.01 [-0.14, 0.12] | 0.893 | 0.14 [-0.00, 0.27] | **0.050** | 0.25 [0.13, 0.36] | **2.71e-05** | 1649 |
| SPON2 | **2.36e-05** | -0.01 [-0.13, 0.10] | 0.810 | -0.02 [-0.15, 0.11] | 0.776 | 0.12 [-0.03, 0.26] | 0.112 | 0.30 [0.17, 0.44] | **6.35e-06** | 1662 |
| PAI | **3.44e-05** | -0.15 [-0.27, -0.02] | **0.019** | 0.00 [-0.13, 0.14] | 0.974 | 0.15 [-0.01, 0.30] | 0.061 | 0.20 [0.07, 0.34] | **0.003** | 1649 |
| TNFR1 | **4.93e-04** | -0.04 [-0.16, 0.08] | 0.527 | 0.02 [-0.12, 0.16] | 0.781 | 0.09 [-0.05, 0.24] | 0.222 | 0.26 [0.13, 0.40] | **8.16e-05** | 1649 |
| FABP4 | **6.53e-04** | -0.02 [-0.12, 0.07] | 0.640 | 0.01 [-0.09, 0.11] | 0.846 | 0.06 [-0.05, 0.17] | 0.300 | 0.21 [0.11, 0.31] | **6.83e-05** | 1649 |
| FAS | **7.35e-04** | -0.12 [-0.26, 0.01] | 0.068 | 0.00 [-0.14, 0.15] | 0.957 | 0.10 [-0.09, 0.28] | 0.310 | 0.26 [0.10, 0.43] | **1.44e-03** | 1649 |
| RARRES2 | **0.002** | -0.09 [-0.21, 0.03] | 0.132 | -0.07 [-0.19, 0.06] | 0.299 | 0.06 [-0.07, 0.20] | 0.373 | 0.16 [0.04, 0.28] | **0.007** | 1649 |
| CCL15 | **0.003** | -0.06 [-0.18, 0.07] | 0.380 | 0.11 [-0.04, 0.25] | 0.156 | 0.02 [-0.12, 0.17] | 0.743 | 0.23 [0.09, 0.38] | **1.33e-03** | 1649 |
| LEP | **0.005** | 0.01 [-0.07, 0.09] | 0.866 | 0.02 [-0.07, 0.10] | 0.700 | 0.04 [-0.06, 0.13] | 0.433 | 0.15 [0.07, 0.24] | **3.76e-04** | 1662 |
| HAOX1 | **0.008** | -0.02 [-0.14, 0.11] | 0.806 | -0.00 [-0.14, 0.13] | 0.990 | 0.06 [-0.09, 0.20] | 0.439 | 0.27 [0.11, 0.43] | **7.13e-04** | 1662 |
| LDLreceptor | **0.008** | -0.14 [-0.26, -0.01] | **0.031** | -0.08 [-0.23, 0.07] | 0.288 | 0.09 [-0.06, 0.24] | 0.237 | 0.09 [-0.05, 0.23] | 0.187 | 1649 |
| PLC | **0.033** | -0.08 [-0.22, 0.06] | 0.256 | 0.01 [-0.12, 0.13] | 0.914 | 0.02 [-0.10, 0.15] | 0.719 | 0.15 [0.04, 0.27] | **0.010** | 1649 |
| IGFBP7 | **0.035** | -0.06 [-0.18, 0.07] | 0.374 | -0.03 [-0.18, 0.12] | 0.696 | 0.05 [-0.09, 0.18] | 0.504 | 0.21 [0.05, 0.37] | **0.009** | 1649 |
| FGF21 | 0.090 | -0.03 [-0.15, 0.09] | 0.618 | -0.08 [-0.22, 0.06] | 0.278 | 0.10 [-0.04, 0.25] | 0.162 | 0.13 [-0.02, 0.27] | 0.092 | 1662 |
| CTSD | 0.127 | -0.01 [-0.13, 0.12] | 0.904 | 0.01 [-0.13, 0.15] | 0.920 | 0.09 [-0.05, 0.22] | 0.216 | 0.16 [0.02, 0.30] | **0.023** | 1649 |
| FGF23 | 0.133 | -0.09 [-0.23, 0.04] | 0.160 | -0.11 [-0.24, 0.03] | 0.121 | -0.03 [-0.17, 0.11] | 0.722 | 0.08 [-0.06, 0.23] | 0.259 | 1662 |
| CTSZ | 0.330 | -0.01 [-0.14, 0.11] | 0.824 | 0.01 [-0.12, 0.15] | 0.864 | 0.05 [-0.09, 0.20] | 0.475 | 0.14 [-0.00, 0.28] | 0.056 | 1649 |

CCL15: C-C motif chemokine 15, CTSD: cathepsin D, CTSZ: cathepsin Z, FAS: tumor necrosis factor receptor superfamily member 6, FABP4: fatty acid-binding protein, adipocyte, FGF21: fibroblast growth factor 21, FGF23: fibroblast growth factor 23, HAOX1: hydroxyacid oxidase 1, IGFBP7: insulin-like growth factor-binding protein 7, LEP: leptin, LDLreceptor: low-density lipoprotein receptor, PAI-1: plasminogen activator inhibitor 1, PLC: perlecan, RARRES2: retinoic acid receptor protein 2, SPON2: spondin-2, tPA: tissue-type plasminogen activator. TNFR1: tumor necrosis factor receptor 1.

**Table S8. Full name of proteins added to the heatmap (Fig. 4).**

| ADM | Adrenomedullin |
| --- | --- |
| ALCAM | CD166 antigen |
| ANGPTL1 | Angiopoietin-related protein 1 |
| ANGPTL7 | Angiopoietin-related protein 7 |
| APN | Aminopeptidase N |
| BAG6 | Large proline-rich protein BAG6 |
| BMP6 | Bone morphogenetic protein 6 |
| CCL15 | C-C motif chemokine 15 |
| CCL16 | C-C motif chemokine 16 |
| CCL3 | C-C motif chemokine 3 |
| CD79B | B-cell antigen receptor complex-associated protein beta chain |
| CDH5 | Cadherin-5 |
| CDHR5 | Cadherin-related family member 5 |
| CEACAM8 | Carcinoembryonic antigenrelated cell adhesion molecule 8 |
| CLMP | CXADR-like membrane protein |
| CLSTN2 | Calsyntenin-2 |
| CNTN1 | Contactin-1 |
| COL1A1 | Collagen alpha-1(I) chain |
| CPA1 | Carboxypeptidase A1 |
| CPB1 | Carboxypeptidase B |
| CSTB | Cystatin-B |
| CTSD | Cathepsin D |
| CTSZ | Cathepsin Z |
| CXCL16 | C-X-C motif chemokine 16 |
| DLK1 | Protein delta homolog 1 |
| EGFR | Epidermal growth factor receptor |
| FABP2 | Fatty acid-binding protein, intestinal |
| FABP4 | Fatty acid-binding protein, adipocyte |
| FAM3C | Protein FAM3C |
| FAS | Tumor necrosis factor receptor superfamily member 6 |
| FCRL1 | Fc receptor-like protein 1 |
| FGF21 | Fibroblast growth factor 21 |
| FGF23 | Fibroblast growth factor 23 |
| FS | Follistatin |
| GDF15 | Growth/differentiation factor 15 |
| GH | Growth hormone |
| GT | Gastrotropin |
| Gal3 | Galectin-3 |
| Gal4 | Galectin-4 |
| Gal9 | Galectin-9 |
| HAOX1 | Hydroxyacid oxidase 1 |
| IDUA | Alpha-L-iduronidase |
| IGFBP1 | Insulin-like growth factor-binding protein 1 |
| IGFBP2 | Insulin-like growth factor-binding protein 2 |
| IGFBP7 | Insulin-like growth factor-binding protein 7 |
| IL18BP | Interleukin-18-binding protein |
| IL1RL2 | Interleukin-1 receptor-like 2 |
| IL1RT2 | Interleukin-1 receptor type 2 |
| IL1ra | Interleukin-1 receptor antagonist protein |
| IL2RA | Interleukin-2 receptor subunit alpha |
| IL6 | Interleukin-6 |
| KIM1 | Kidney Injury Molecule |
| KLK6 | Kallikrein-6 |
| LDL receptor | Low-density lipoprotein receptor |
| LEP | Leptin |
| LPL | Lipoprotein lipase |
| LRIG1 | Leucine-rich repeats and immunoglobulin-like domains protein 1 |
| MCP1 | Monocyte chemotactic protein 1 |
| MEPE | Matrix extracellular phosphoglycoprotein |
| MMP7 | Matrix metalloproteinase-7 |
| OPG | Osteoprotegerin |
| PAI | Plasminogen activator inhibitor 1 |
| PARP1 | Poly [ADP-ribose] polymerase 1 |
| PCSK9 | Proprotein convertase subtilisin/kexin type 9 |
| PDL2 | Programmed cell death 1 ligand 2 |
| PECAM1 | Platelet endothelial cell adhesion molecule |
| PGF | Placenta growth factor |
| PGLYRP1 | Peptidoglycan recognition protein 1 |
| PI3 | Elafin |
| PLC | Perlecan |
| PON3 | Paraoxonase |
| PRSS8 | Prostasin |
| RARRES2 | Retinoic acid receptor responder protein 2 |
| SCF | Stem cell factor |
| SELP | P-selectin |
| SEMA3F | Semaphorin-3F |
| SIGLEC7 | Sialic acid-binding Ig-like lectin 7 |
| SPON2 | Spondin-2 |
| SUMF2 | Sulfatase-modifying factor 2 |
| TFF3 | Trefoil factor 3 |
| TFPI | Tissue factor pathway inhibitor |
| TIE2 | Angiopoietin-1 receptor |
| TLT2 | Trem-like transcript 2 protein |
| TNFR1 | Tumor necrosis factor receptor 1 |
| TNFR2 | Tumor necrosis factor receptor 2 |
| TNFRSF10A | Tumor necrosis factor receptor superfamily member 10A |
| TNFRSF11A | Tumor necrosis factor receptor superfamily member 11A |
| TNFRSF13B | Tumor necrosis factor receptor superfamily member 13B |
| TNFRSF14 | Tumor necrosis factor receptor superfamily member 14 |
| TNFSF13B | Tumor necrosis factor ligand superfamily member 13B |
| TRAILR2 | TNF-related apoptosis-inducing ligand receptor 2 |
| TRAP | Tartrate-resistant acid phosphatase type 5 |
| TSHB | Thyrotropin subunit beta |
| UPAR | Urokinase plasminogen activator surface receptor |
| tPA | Tissue-type plasminogen activator |
| vWF | von Willebrand factor |
